# Note from the editors: Open access and sound science for rapid public health action

**DOI:** 10.2807/1560-7917.ES.2019.24.2.1901101

**Published:** 2019-01-10

**Authors:** Eurosurveillance editorial team

**Affiliations:** 1 European Centre for Disease Prevention and Control (ECDC), Stockholm, Sweden

**Keywords:** public health, Europe, artificial intelligence, GDPR, CONSORT, PRISMA

In 2018, public health experts and scientists concerned with the epidemiology and control of infectious diseases in Europe continued to be busy handling outbreaks and (re)-emerging infectious diseases; however, in contrast to recent years, there was no single overriding public health event that caught global attention. Nonetheless, there were developments that kept experts alert and that we editors followed with great interest.

Artificial intelligence (AI) continued to gain momentum and was widely acknowledged as an evolution that will fundamentally affect our societies and the ways that we generate knowledge. In the field of infectious disease, concrete examples of self-learning applications with some freedom of decision-making are still scarce. This was one of the messages from the 2018 *Eurosurveillance* seminar at ESCAIDE, Artificial intelligence (AI) in epidemiology: a reality in 2018? Discussions at this event also brought forward that now is a good time to tackle basic questions on ethics, infrastructure and training needs for epidemiologists and (public health) microbiologists related to AI. Such questions need to be addressed in an interdisciplinary collaboration with computer scientists, ethicists, social scientists and many others. We at *Eurosurveillance* will keep an eye on further developments on this topic, and we welcome the submission of articles in which authors share concrete examples of applied AI in public health and infectious disease epidemiology and surveillance.

The editorial team continuously strives to increase transparency of reporting, as well as the quality of articles published in *Eurosurveillance*. In 2018, we fine-tuned the authors’ instructions for outbreak articles. New instructions for surveillance articles were developed in collaboration with the editorial board and will be posted online soon. They should streamline and thus improve the presentation of surveillance data to boost the impact of this important feature of *Eurosurveillance*. From the beginning of 2019, *Eurosurveillance* has mandated the depositing of sequence data in open access public repositories ahead of submission, as well as the inclusion of ethical statements in regular articles. The journal’s editorial policy has endorsed the use of several reporting guidelines for many years, and starting in 2019 we will mandate that new submissions include checklists for certain article types: the PRISMA checklist for systematic reviews, the CHEERS checklist for health economic studies and the CONSORT checklist for clinical trials. While we strongly recommend that authors follow STROBE guidelines for the reporting of observational studies, we do not mandate the submission of STROBE checklists at present.

Support from reviewers, editorial board members, colleagues, our publisher the European Centre for Disease Prevention and Control (ECDC) and ECDC’s Director, as well as many others, has been invaluable in 2018. We are immensely grateful for the strategic and scientific advice, moral and day-to-day support and sustained funding that allows us to publish and disseminate information we deem to be sound science capable of informing public health decision-making.

Peer-review offers a great opportunity to engage experts in improving the quality of scientific work by adding new perspectives and insight, when reviews are conducted thoroughly and respectfully. We are aware that peer-review has been criticised as ineffective and biased by parts of the scientific community. Still, we believe it is a learning opportunity for both those giving feedback and those receiving it, and we editors who moderate this process profit considerably from everyone who gives their time and shares their expertise to guide us and authors. To support reviewers in their assessment of the completeness and comprehensiveness of manuscripts, we will send the filled-in checklists submitted by authors together with the manuscripts for review, and we hope that reviewers will find this useful.

Our means to express our gratitude for support are limited. Traditionally, we publish a list with the names of all peer-reviewers at the beginning of each year to thank them. In 2018, nearly 500 individuals supported us with formal reviews [1], and we would like to extend our thanks to all those who may remain unnamed but who nonetheless provided helpful advice. In order to further recognise our peer-reviewers, we will send certificates to experts who completed more than one review in 2018. We are also happy to confirm reviewing activities on recognised platforms such as Publons [2], upon request. Please do not hesitate to contact the editorial office if you need a certificate or confirmation in another way.

In 2018, we published 182 articles (52 Rapid communications, 115 regular articles, 15 other items such as editorials, letters and meeting reports). The 2018 acceptance rate of 26% was similar to previous years and, while we received submissions from around the world, the selection of articles was guided by relevance for public health in Europe and thus the vast majority of published articles were from Europe. Our Rapid communications, published within 2 to 3 weeks of submission, reported on ongoing or emerging threats to support rapid public health action, as we have done for many years. We covered events such as the nationwide outbreak of *Salmonella* Agona associated with internationally distributed infant milk products in France [3], the unusually early start of West Nile virus transmission season in Europe [4] and the upsurge of different enteroviruses associated with severe illness in some countries [5-7], and also published timely communications about influenza vaccine effectiveness [8-11].

We published several topical and special issues that collated data and evidence to support communication on specific topics, in some instances for particular health days or weeks. These issues covered important aspects of vaccination, HIV/AIDS and antimicrobial resistance, topics that continue to be high on our agenda. We hope that the articles we have published—such as those on the results from the 2016 to 2017 European point prevalence survey on antimicrobial use and healthcare-associated infections in acute and long-term care facilities [12]—serve as quality evidence to support effective public health decision-making.

The 2018 special issues resulting from dedicated calls for papers were ‘Screening and prevention of infectious diseases in newly arrived migrants in Europe’ and, in December, the first part of a special issue demonstrating how novel approaches in molecular diagnostics support traditional epidemiology and public heath decision-making, the second part of which will follow at the end of January 2019. In February, we will launch a call for papers on the use of point of impact testing (POIT)/point of care testing (POCT) and self-testing in surveillance and epidemiology.

Various metrics showed that *Eurosurveillance* remained among the leading journals in its field in 2018. The journal’s most recent impact factor, released in mid-2018, was 7.1, ranking *Eurosurveillance* fifth in the category Infectious Diseases. The journal remained in the first quartile (for all categories listed) in the SCImago Journal Rank and Google Scholar metrics continued to be equally favourable. The Scopus-based CiteScore for *Eurosurveillance* improved from 3.7 to 5.0 and the CiteScore Percentile went up to 98%, corresponding to rank six among 478 journals in the category Medicine: Public Health, Environmental and Occupational Health.

Of relevance to our authors, reviewers and subscribers, 2018 also brought the new General Data Protection Regulation (GDPR) for Europe [13]. The GDPR caused concern for some, but reassured others that their privacy will be better protected. *Eurosurveillance* editors have long been sensitive to the need to protect the privacy of individuals and most GDPR requirements were already being followed before it came into effect.

In light of the growing number of funders requesting instant open access for (community)-funded science, we would like to remind our authors and readers that *Eurosurveillance* is a well-recognised, open-access journal that provides a European platform for health professionals to share quality scientific findings in infectious disease epidemiology, prevention and control.

At *Eurosurveillance* we nurture close ties with our collaborators, authors, board members and colleagues at ECDC, and we enjoy this part of our work. It helps us develop the journal and identify topics of interest. Moreover, we gain insight into public health needs, which is crucial for our aim to be the authoritative and representative public health voice of the communicable disease community in Europe and beyond. Towards this objective, we aspire to provide facts and guidance for health professionals and decision-makers, to facilitate the implementation of effective prevention and control measures, and to support the preparedness and response to health threats in Europe through the rapid dissemination of high-quality, authoritative scientific information on relevant outbreaks or emergency situations. We look forward to fulfilling our aims in 2019, jointly with our contributors and many other supporters, and we wish all of them a productive and good year!
